# Metabolomics of cerebrospinal fluid reveals candidate diagnostic biomarkers to distinguish between spinal muscular atrophy type II and type III

**DOI:** 10.1111/cns.14718

**Published:** 2024-04-14

**Authors:** Mengnan Lu, Xueying Wang, Na Sun, Shaoping Huang, Lin Yang, Dan Li

**Affiliations:** ^1^ Department of Pediatrics the Second Affiliated Hospital of Xi'an Jiaotong University Xi'an Shaanxi China

**Keywords:** cerebrospinal fluid, diagnostic biomarkers, metabolomics, spinal muscular atrophy

## Abstract

**Aims:**

Classification of spinal muscular atrophy (SMA) is associated with the clinical prognosis; however, objective classification markers are scarce. This study aimed to identify metabolic markers in the cerebrospinal fluid (CSF) of children with SMA types II and III.

**Methods:**

CSF samples were collected from 40 patients with SMA (27 with type II and 13 with type III) and analyzed for metabolites.

**Results:**

We identified 135 metabolites associated with SMA types II and III. These were associated with lysine degradation and arginine, proline, and tyrosine metabolism. We identified seven metabolites associated with the Hammersmith Functional Motor Scale: 4‐chlorophenylacetic acid, adb‐chminaca,(+/−)‐, dodecyl benzenesulfonic acid, norethindrone acetate, 4‐(undecan‐5‐yl) benzene‐1‐sulfonic acid, dihydromaleimide beta‐d‐glucoside, and cinobufagin. Potential typing biomarkers, N‐cyclohexylformamide, cinobufagin, cotinine glucuronide, N‐myristoyl arginine, 4‐chlorophenylacetic acid, geranic acid, 4‐(undecan‐5‐yl) benzene, and 7,8‐diamino pelargonate, showed good predictive performance. Among these, N‐myristoyl arginine was unaffected by the gene phenotype.

**Conclusion:**

This study identified metabolic markers are promising candidate prognostic factors for SMA. We also identified the metabolic pathways associated with the severity of SMA. These assessments can help predict the outcomes of screening SMA classification biomarkers.

## Graphical abstract

We have demonstrated metabolic markers in CSF associated with the classification of SMA in children, including 4‐chlorophenylacetic acid, sodium dodecyl benzenesulfonate, and cinobufagin. We also identified the metabolic pathways associated with the severity of SMA, including lysine degradation, arginine metabolism, and tyrosine metabolism.

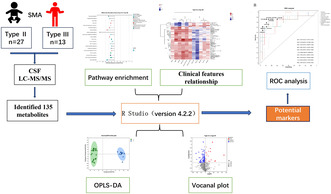

## 1 INTRODUCTION

Spinal muscle atrophy (SMA) is a neurogenic muscle disease characterized by motor neuron degeneration and severe muscular atrophy.^1^ SMA is an autosomal recessive disease caused by a mutation in the surviving motor neuron gene (*SMN1*) on the long arm of chromosome 5 (5q13.1). Homozygous deletion of exon 7 of *SMN1* is present in more than 90% of the patients with SMA, and a few patients carry point mutations.^2^ The *SMN2* gene is highly homologous to the *SMN1* gene, differing from it by only five nucleotides. One variant in exon 7 of *SMN2*, c.840C > T, results in the production of a truncated, nonfunctional, and rapidly degraded unstable protein (SMN‐Δ7).^3^ Approximately 10% of *SMN2* transcripts produce a full‐length protein, providing patients with SMA with a small amount of SMN protein to maintain spinal cord motor neurons.^4^ SMA occurs in 1/6000–1/10,000 newborns, and the carrier frequency of SMA is approximately 1 in 40–50 people in the general population.^5^ SMA can be divided into five types according to genetic test results and clinical manifestations, with type I accounting for the largest proportion of cases.^6^

Since the approval of nusinersen as the first drug for SMA by the Food and Drug Administration (FDA),^7^ three drugs have been adopted for SMA treatment globally. For patients with types I and II, there is widespread agreement that early presymptomatic treatment is essential.^8^ However, for patients with type III or type IV, the appropriate time for presymptomatic treatment remains controversial. Metabolomics is widely used in research because metabolites have significant effects on biological systems.^9^ Cerebrospinal fluid (CSF) plays several key roles in the central nervous system (CNS), including structural protection, immune support, and metabolic homeostasis.^10^ The CSF offers an avenue for detecting various pathophysiological changes in the CNS, including metabolic changes.^11^ Metabolomics is a powerful tool for studying the CNS,^12^ including neuroinflammation,^13^ brain tumors,^14^ neurodegeneration,^15^ psychiatric disorders,^16^ and epilepsy.^17^ The diagnostic classification of SMA relies on its clinical presentation, which is often delayed and cannot be relied upon for making timely assessments. Therefore, accurate biomarkers are required for the prediction of SMA type, prognosis, drug response, and overall treatment effects.^18^ Previous studies have performed metabolites on CSF from adult patients with SMA III and observed a correlation with CSF metabolites.^19^ However, little research has been conducted on metabolites in CSF from children with SMA. In the present study, baseline data from 40 children with SMA types II and III were analyzed, and CSF samples were collected for nontargeted metabolomics testing at the time of first treatment. The aim of the present study was to enhance our understanding of the pathological process of SMA in patients with clinically differentiated SMA and to identify metabolic markers in the CSF of children with SMA types II and III.

## 2 MATERIALS AND METHODS

### 2.1 Study participants

This study was approved by the Ethics Committee of the Second Affiliated Hospital of Xi'an Jiaotong University (2022 Ethics Approval—Research No. 038). Overall, 40 children with SMA were recruited for this study. All participants were from the SMA Treatment Center of the Xi'an Jiaotong University Second Affiliated Hospital. SMA was diagnosed using a combination of genetic reports and clinical presentations by two experienced senior physicians. When the two doctors agreed on the diagnosis, the SMA classification was determined; when there was no agreement, a third experienced doctor evaluated the diagnosis. The inclusion criteria were as follows^20^: (1) age ≥ 24 months and ≤168 months; (2) clinical symptoms and genetic testing reports that met the diagnostic criteria for 5qSMA; (3) availability of complete clinical data. Exclusion criteria were as follows: (1) severe infections, liver and kidney failure, and fracture occurrence during the study; (2) age younger than 24 months; and (3) genetic results unavailable or not supporting 5qSMA diagnosis.

Families of all participants included in this study provided informed consent, as did children older than 8 years, all of whom were given comprehensive explanations about the study. All participants underwent tests for clinical signs and symptoms, CSF examination, spinal radiography, and genetic testing. Reports were collected at their first visit for baseline assessment. The Hammersmith Functional Motor Scale Expanded (HFMSE) assesses the functional motor abilities of patients with SMA who can sit and walk. All children were evaluated using the HFMSE. CSF was stored for follow‐up testing in all patients at the time of the first intranasal administration of nusinersen. Prior to CSF sample collection, none of the patients received any treatment for SMA, including nusinersen, risdiplam, salbutamol, or any other treatment.

### 2.2 CSF sample preparation

All CSF samples were centrifuged immediately after collection (800 × *g*; 5 min) and stored in centrifuge tubes as 500 μL aliquots. Samples were stored at −80°C and tested together after all samples were collected. To prepare the CSF samples, they were carefully thawed on ice. Briefly, 100 μL of liquid sample was added to a 1.5‐mL centrifuge tube with 400 μL solution [acetonitrile: methanol in 1:1 (v:v) ratio] containing 0.02 mg/mL internal standard (L‐2‐chlorophenylalanine) to extract metabolites. The samples were mixed by vortexing for 30 s and sonicated at a low temperature for 30 min (5°C, 40 KHz). The samples were incubated at −20°C for 30 min to precipitate the proteins. Then, the samples were centrifuged for 15 min (4°C, 13000 × *g*). The supernatant was removed and dried under nitrogen. The sample was then re‐solubilized with 100 μL solution (acetonitrile: water = 1:1) and extracted by low‐temperature ultrasonication for 5 min (5°C, 40 KHz), followed by centrifugation at 13,000 × *g* and 4°C for 10 min. The supernatant was transferred to sample vials for liquid chromatography–tandem mass spectrometry (LC–MS/MS) analysis.^21^

As part of the system conditioning and quality‐control processes, a pooled quality‐control sample (QC) was prepared by mixing equal volumes of all samples. The QC samples were disposed of and tested in the same manner as the analytical samples. This helped us represent the whole sample set, which was injected at regular intervals (every 5–15 samples) to monitor the stability of the analysis.

### 2.3 Ultra‐performance LC–MS/MS analysis

The LC–MS/MS analysis was conducted on a Thermo UHPLC‐Q Exactive system equipped with an ACQUITY HSS T3 column (100 mm × 2.1 mm i.d., 1.8 μm; Waters, USA) at Majorbio Bio‐Pharm Technology Co., Ltd. (Shanghai, China). The mobile phase comprised 0.1% formic acid in water: acetonitrile (95:5%, v/v) (solvent A) and 0.1% formic acid in acetonitrile and isopropanol: water (47.5:47.5, v/v) (solvent B). The flow rate was 0.40 mL/min, and the column temperature was 40°C. The injection volume was 3 μL.

### 2.4 Data processing

Pre‐processing of raw LC/MS data was performed using Progenesis QI software (Waters Corporation, Milford, USA). The metabolites were identified by searching the HMDB (http://www.hmdb.ca/), Metlin (https://metlin.scripps.edu/), and Majorbio databases.

Data were analyzed using R Studio (Version 4.2.2). The R package, “ropls” (Version 1.6.2), was used to perform orthogonal least partial squares discriminant analysis (OPLS‐DA) and 7‐cycle interactive validation for evaluating the model's stability. Variable importance in the projection (VIP) was obtained using the OPLS‐DA model, and the p value was determined using the Student's *t*‐test. Metabolites with *p* values < 0.05, false discovery rate (FDR) < 0.2, and VIP values > 1 were considered statistically significant in this study.^22^, ^23^ Differential metabolites between the two groups were mapped to their biochemical pathways through metabolic enrichment and pathway analysis using the KEGG database (http://www.genome.jp/kegg/). The R packages “heatmap,” “volcano,” “pROC,” and “wgcna” were used to plot the heatmap, volcano plot, and receiver operating characteristic (ROC) curve and perform WGCNA, respectively.

### 2.5 Statistical data analysis

Statistical analyses were performed using IBM SPSS Statistics version 24 (IBM Corp., Armonk, NY, USA). All data were subjected to the Shapiro–Wilk test for normality. Data that exhibited a normal distribution are expressed as mean ± standard deviation, and data that deviate from normal distribution are expressed as median (interquartile range). Comparisons between two groups of normally distributed data were conducted using the *t*‐test, while the Mann–Whitney rank test was used for comparisons between two groups of non‐normally distributed data. Chi‐squared test was used to compare rates between the two groups. Statistical significance was set at *p* < 0.05.

## 3 RESULTS

### 3.1 Clinical characteristics of the participants

Overall, 40 children diagnosed with SMA were enrolled in this study, including 27 with type II SMA and 13 with type III SMA (Table 1). There were no significant differences in age, sex, scoliosis, genetic test results, or biochemical characteristics of the CSF between the two groups (*p* > 0.05). However, there were significant differences in age of onset. In addition, the HFMSE score was significantly different between the two groups (*p* < 0.001). Among the children with type II SMA in this study, three had compound heterozygous mutations, two had the c.863G > T mutation, and one had the c.569G > A mutation. Overall, four children with type III SMA had compound heterozygous mutations: one had the c.863G > T mutation, two brothers had the c.400G > A mutation, and one had the c.*2 A > T mutation. The proportion of compound heterozygous mutations was approximately 17.5%. There were two reasons for this phenomenon. Firstly, children with SMA younger than 24 months were excluded from the study due to the age restrictions imposed by the HFMSE scale. Secondly, the small number of cases (*n* = 40) contributed to selection bias in this study.

**TABLE 1:** Clinical characteristics of participants.

Characteristics	II	III	*p* Value
*N*	27	13	
Age (months), median (interquartile range)	64 (50, 108)	94 (57.5, 153)	0.1887
Age of onset (months), median (interquartile range)	12 (8, 15)	22 (18, 33)	<0.0001
Sex, *n* (%)			0.564
Girls	13 (32.5%)	5 (12.5%)	
Boys	14 (35.0%)	8 (20.0%)	
Scoliosis, *n* (%)			0.056
Yes	17 (42.5%)	4 (10.0%)	
No	10 (25.0%)	9 (22.5%)	
HFMSE, mean ± SD	13.37 ± 11.526	45.308 ± 16.555	<0.001
*SMN1*			0.125
Homozygous deletion	24 (60%)	9 (22.5%)	
Compound heterozygous mutation	3 (7.5%)	4 (10%)	
Sitter	27 (67.5%)	1 (2.5%)	
Walker	0 (0.0%)	12 (30.0%)	
*SMN2* copy number *n* (%)			0.160
3 copy	20 (50%)	11 (27.5%)	
2 copy	7 (17.5%)	1 (2.5%)	
4 copy	0 (0%)	1 (2.5%)	
CSF_LDH (U/L)	18.556 ± 5.071	16.615 ± 6.449	0.306
CSF_Pro (g/L)	0.272 ± 0.096	0.280 ± 0.107	0.786
CSF_Glu (mmol/L)	3.496 ± 0.270	3.4792 ± 0.352	0.869
CSF_Cl (mmol/L)	127.62 ± 2.494	127.72 ± 1.503	0.898

Abbreviations: Cl, chloride; CSF, cerebrospinal fluid; Glu, glucose; HFMSE, Hammersmith Functional Motor Scale Expanded; II, spinal muscular atrophy type II; III, spinal muscular atrophy type III; LDH, lactate dehydrogenase; Pro, protein.

### 3.2 Metabolic profiling of CSF from SMA samples of different types

**FIGURE 1:**
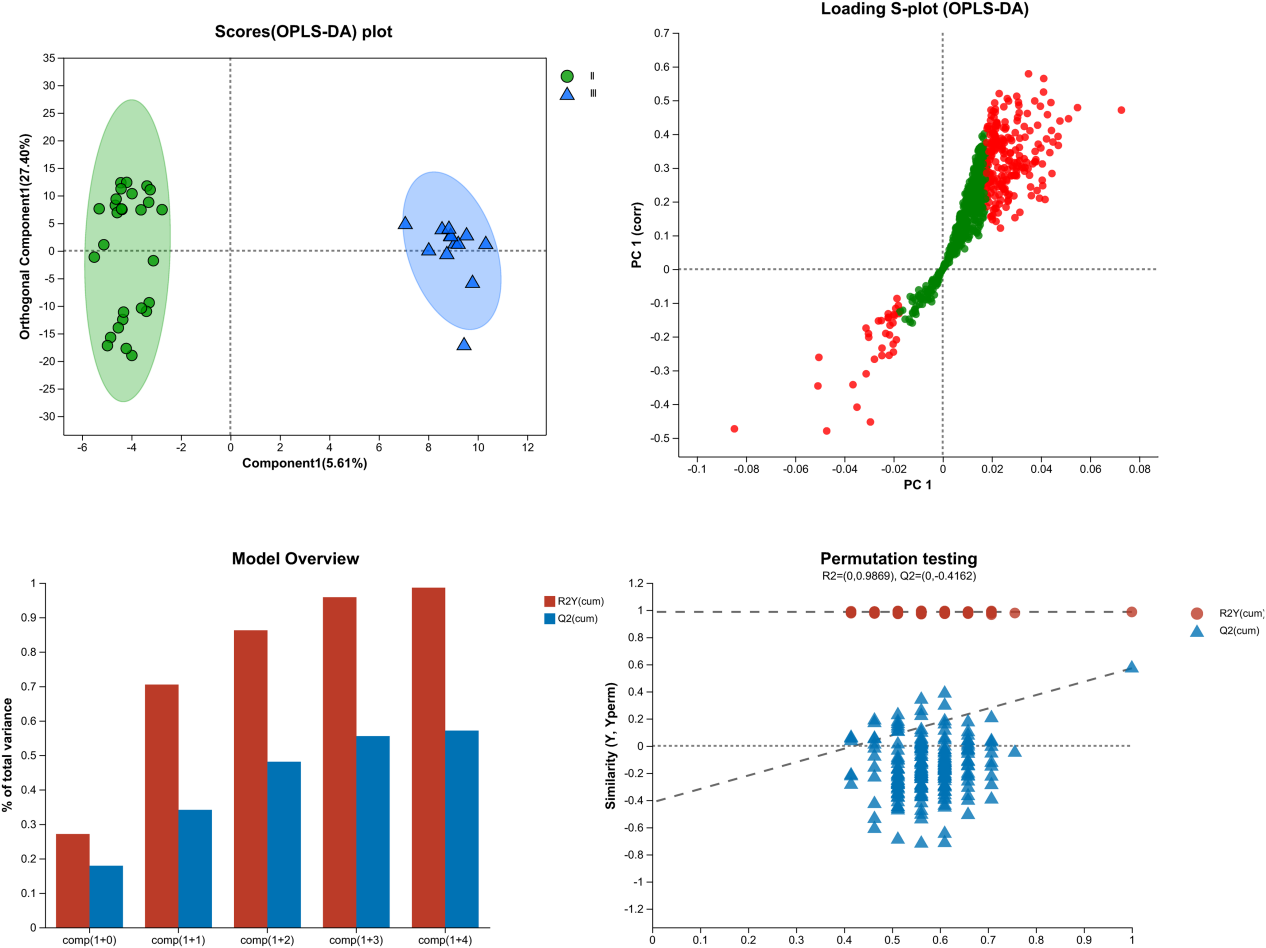
Orthogonal partial least squares discriminant analysis (OPLS‐DA) score scatter plot of spinal muscular atrophy (SMA) types II and III.

To characterize the metabolomic features of the different types of SMAs, a comparative study involving patients with SMA type II and SMA type III was performed. To comprehensively identify differential metabolites, we constructed an OPLS‐DA model (R2Y = 0.987, Q2 = −0.416). The CSF metabolites of children with SMA type II could be differentiated from those of children with SMA type III (Figure 1).

**FIGURE 2:**
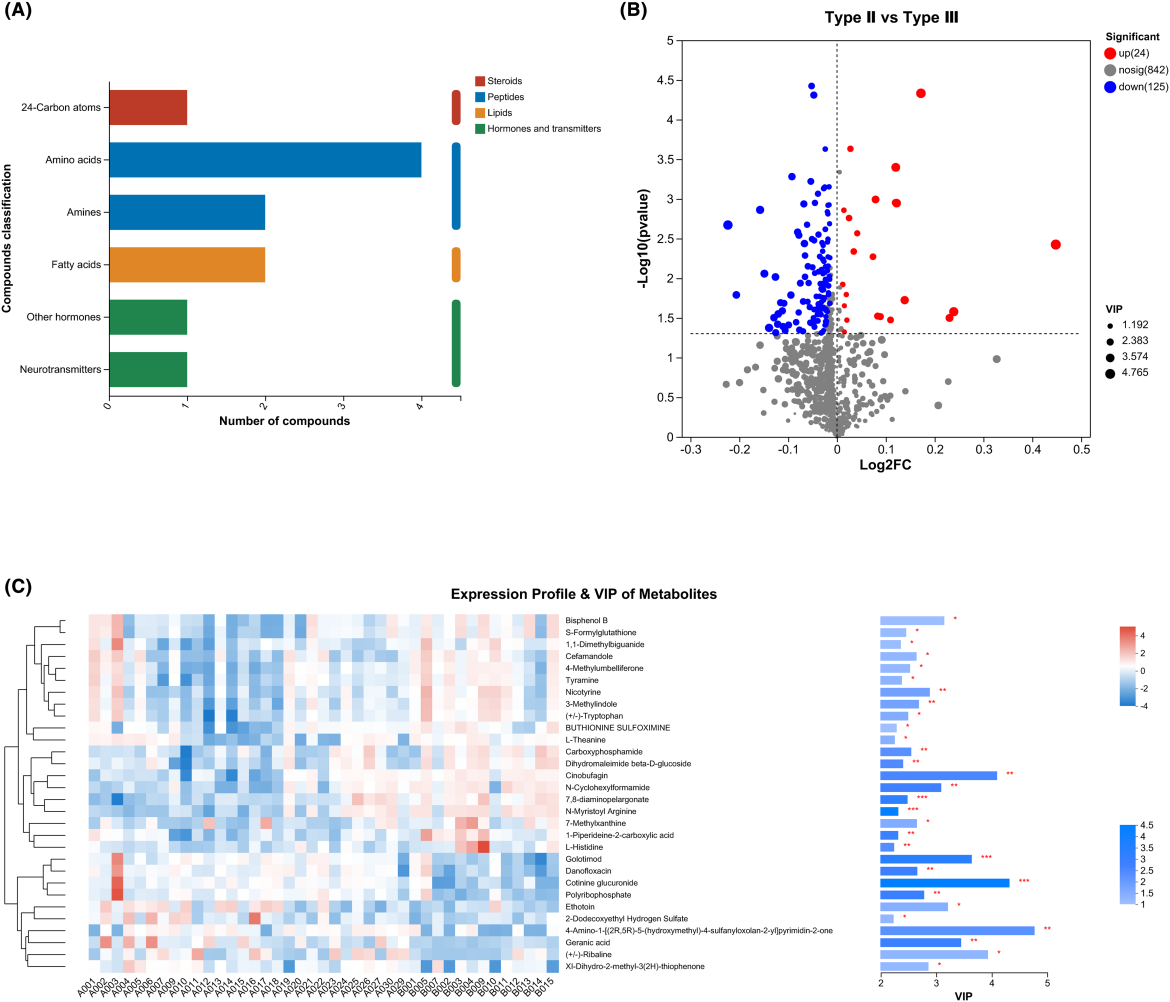
Different metabolites in the cerebrospinal fluid (CSF) of patients with spinal muscular atrophy (SMA) type II and SMA type III. (A) Classes of metabolites in CSF. (B) Volcano plot of metabolites in CSF. (C) Top‐30 metabolites with differences based on a comprehensive assessment of *p* value and variable importance in the projection (VIP).

This study identified five classes of metabolites in the CSF: steroids, peptides, lipids, hormones, and transmitters (Figure 2A). A total of 135 metabolites with *p* < 0.05, FDR < 0.2, and VIP > 1 were identified using the OPLS‐DA model (Figure 2B, Supplementary Information S1). The top‐30 metabolites with differences based on the comprehensive judgment of the *p* value and VIP are shown in Figure 2C.

### 3.3 Influence of genetic phenotype on the different CSF metabolites from patients with SMA type II and type III

**FIGURE 3:**
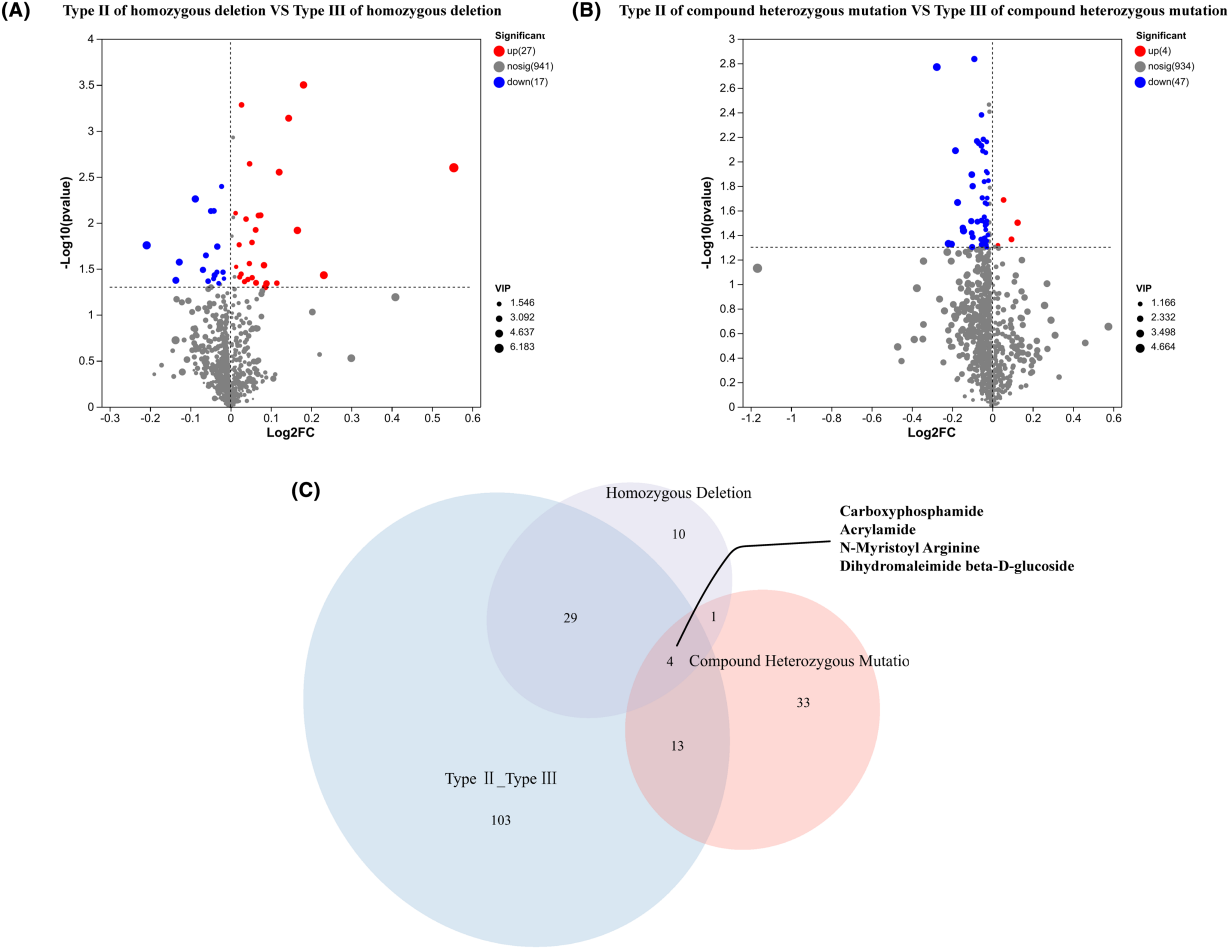
Different metabolites in the cerebrospinal fluid (CSF) of patients with spinal muscular atrophy (SMA) harboring compound heterozygous mutation and homozygous deletion. (A) Differential metabolites between patients with SMA type II harboring homozygous deletion and those with SMA type III harboring homozygous deletion. (B) Differential metabolites between patients with SMA type II harboring compound heterozygous mutation and those with SMA type III harboring compound heterozygous mutation. (C) Venn diagram of differential metabolites. Blue circle: Differential metabolites between SMA type II and type III. Purple circle: Differential metabolites between SMA type II with homozygous deletion and SMA type III with homozygous deletion.

Patients with compound heterozygous mutations were grouped into type II and type III, those with homozygous deletion mutations were grouped into type II and type III, and their respective metabolites were compared separately. Forty‐four metabolites were differentially expressed between type II and type III in patients with homozygous deletion mutations (Figure 3A). In contrast, 51 metabolites were differentially expressed in patients with compound heterozygous mutations. As shown in Figure 3B, only four differentially expressed metabolites overlapped among the three groups of patients, including carboxyphosphamide, acrylamide, N‐myristoyl arginine, and dihydromaleimide beta‐D‐glucoside (Figure 3C).

### 3.4 Enrichment analysis for CSF metabolites

**FIGURE 4:**
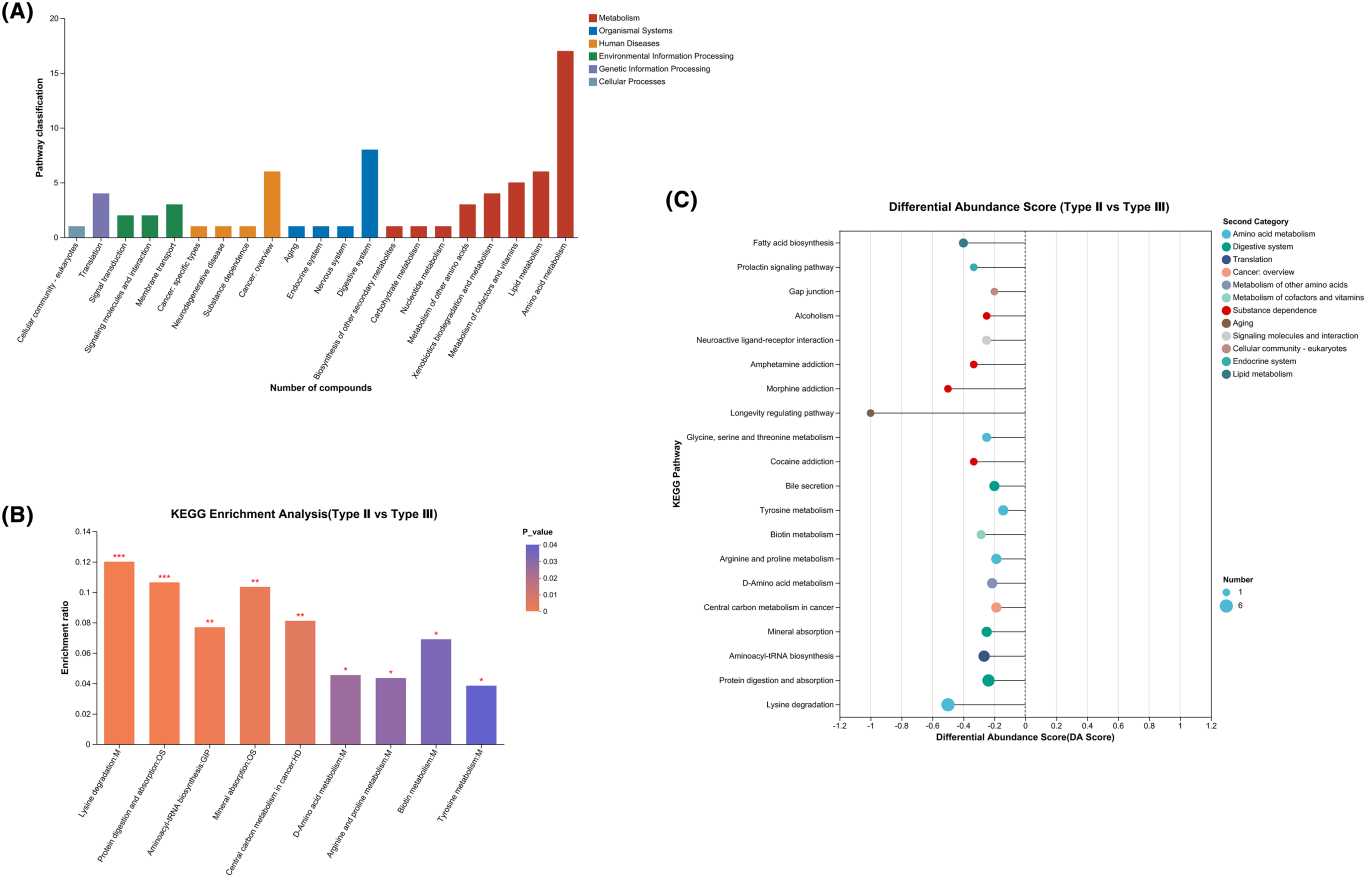
Enrichment analysis of cerebrospinal fluid (CSF) metabolites. (A) Classification of compounds identified by KEGG enrichment analysis. (B) Enrichment analysis of CSF metabolites based on *p* value. (C) Enrichment analysis of CSE metabolites based on abundance score. *p value or FDR < 0.05, **p value or FDR < 0.01, ***p value or FDR < 0.001.

The compounds identified in this study were mainly concentrated in metabolic pathways, and fewer compounds were concentrated in organismal systems, human diseases, and environmental formation processing pathways (Figure 4A). KEGG enrichment analysis showed that CSF metabolites were mainly concentrated in pathways related to lysine degradation, protein digestion and absorption, aminoacyl‐tRNA biosynthesis, mineral absorption, central carbon metabolism in cancer, arginine and proline metabolism, biotin metabolism, D‐amino acid metabolism, and tyrosine metabolism (Figure 4B,C, Supplementary Information S2).

### 3.5 Correlation between CSF metabolites and clinical features

**FIGURE 5:**
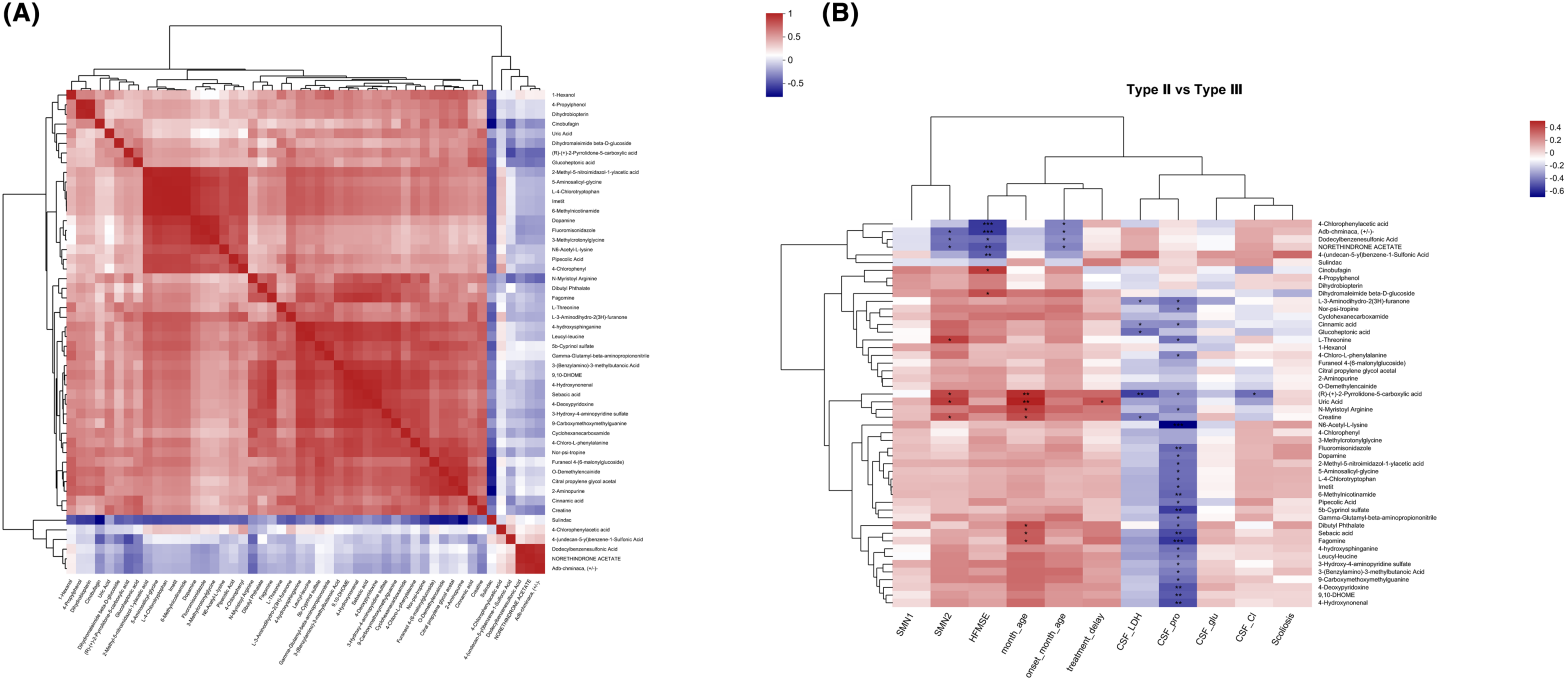
Correlation between CSF metabolites and clinical features. (A) Correlation between metabolites. (B) Correlation between metabolites and clinical features. CSF, cerebrospinal fluid; HFMSE, Hammersmith Functional Motor Scale Expanded; LDH, lumbar disk herniation; *SMN1*, survival of motor neuron 1; *SMN2*, survival of motor neuron 2.

To further clarify whether the metabolites could be used as diagnostic markers to distinguish SMA subtypes, correlation analyses were performed. The correlation between the CSF metabolites is shown in Figure 5A. The correlation between the CSF metabolites and clinical features is shown in Figure 5B (Supplementary Information S3). Notably, dihydromaleimide beta‐D‐glucoside and cinobufagin were positively and significantly correlated with HFMSE (*r* = 0.3502 and 0.3976, respectively; *p =* 0.02 and 0.01, respectively), whereas 4‐chlorophenylacetic acid, adb‐chminaca, dodecyl benzenesulfonic acid, norethindrone acetate, and 4‐(undecan‐5‐yl) benzene‐1‐sulfonic acid were negatively and significantly correlated with HFMSE (*p* < 0.05). Detailed *p* values and *r*‐values are shown in Supplementary Information S3.

### 3.6 ROC analysis

**FIGURE 6:**
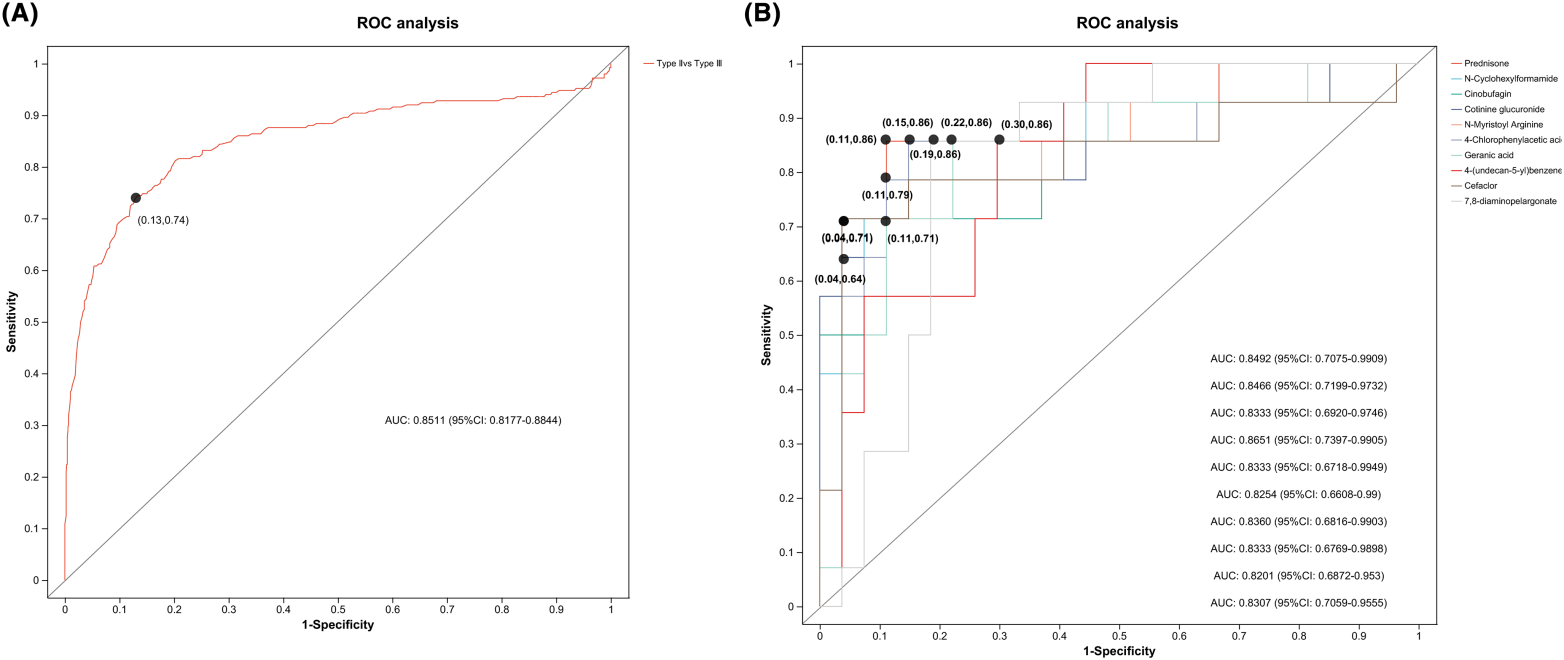
ROC analysis. (A) Multivariate ROC curve analysis. (B) Univariate ROC curve analyses. AUC, area under the curve; ROC, receiver operating characteristic.

To further evaluate the typing prediction performance of the identified metabolites, we performed univariate and multivariate ROC curve analyses to distinguish between children with SMA type II and SMA type III. We first performed a multivariate ROC curve analysis based on all previously identified differential metabolites, with an area under the curve (AUC) value of 0.8511 (95% CI: 0.8177–0.8844), suggesting that differential metabolites could accurately predict SMA classification (Figure 6A). According to the classical univariate ROC curve classification models, 11 metabolites had an AUC ≥ 80% for typing prediction of SMA. The levels of 10 major metabolites—prednisone, N‐cyclohexyl formamide, cinobufagin, cotinine glucuronide, N‐myristoyl arginine, 4‐chlorophenylacetic acid, geranic acid, 4‐(undecan‐5‐yl) benzene, cefaclor, and 7,8‐diamino pelargonate—were plotted (Figure 6B, Supplementary Information S4).

## 4 DISCUSSION

This study provided a comprehensive metabolic assessment of CSF from children with different subtypes of SMA and identified 135 metabolites associated with these subtypes. These metabolites were found to be associated with lysine degradation, arginine and proline metabolism, biotin metabolism, D‐amino acid metabolism, and tyrosine metabolism by bioinformatics analysis. We identified seven metabolites associated with HFMSE, including 4‐chlorophenylacetic acid, adb‐chminaca, dodecyl benzenesulfonic acid, norethindrone acetate, 4‐(undecan‐5‐yl) benzene‐1‐sulfonic acid, dihydromaleimide beta‐d‐glucoside, and cinobufagin. Potential typing biomarkers, N‐cyclohexyl formamide, cinobufagin, cotinine glucuronide, N‐myristoyl arginine, 4‐chlorophenylacetic acid, geranic acid, 4‐(undecan‐5‐yl) benzene, and 7,8‐diamino pelargonate, showed good predictive performance.

By definition, patients with type II SMA show weakness after 6 months of age, and the ability to sit is their most significant motor milestone. Individuals with SMA type III develop symptoms after 18 months of age and gain the ability to walk independently.^24^ However, owing to recall bias, patients and their families are often unable to determine the best exercise state, posing a challenge to clinical diagnostic typing. CSF is essential for the diagnosis of neurological diseases.^25^ In the present study, patients with SMA type III were aged between 18 and 144 months at disease onset. Premature presymptomatic treatment of patients with type III SMA may incur major medical costs and represent a huge financial burden for families. It is widely acknowledged that *SMN2* copy number can predict clinical classification.^26^ However, when *SMN2* copy number is 2 or 3, it does not provide any indication as to whether SMA is type II or type III. Therefore, it is imperative to identify new biomarkers to facilitate the prediction of clinical classifications.

CSF biomarkers assist in the diagnosis of various neurological disorders in children.^27^ In this study, we investigated metabolic markers in the CSF of children with SMA, identifying significantly different metabolites in the CSF of patients with SMA type II and type III. However, since the timing of CSF sample collection was after the clinical confirmation of the diagnosis, these metabolites may not be sufficient to predict clinical typing but could be candidates for predicting the prognosis of SMA.

Lysine is associated with nerve growth factor receptors and promotes the proliferation^28^ and differentiation of neural progenitor cells.^29^ Recent studies have reported that lysine‐less variants of SMA, SMN, and SMNΔ7 proteins are degraded.^30^ Arginine methylation is associated with neurodegeneration^31^ given the toxicity of the protein arginine methyltransferase 6, which enhances polyglutamine‐amplified androgen receptor function and spinal cord and medullary muscle atrophy.^32^ The function of the protein arginine methyltransferase is dysregulated in SMA.^33^ Receptor tyrosine kinases are neurotrophic factor receptors^34^ that are involved in neuromuscular junction signaling. Tyrosine kinases are associated with neurite growth disorders in SMA models and SMA treatment.^35^ Results of our bioinformatics studies showed that lysine degradation, arginine metabolism, and tyrosine metabolism pathways were associated with SMA and differed in the SMA classification.

4‐chlorophenylacetic acid, a derivative of phenylacetate, is useful for the prevention or treatment of estrogen‐sensitive breast cancer^36^, ^37^ and human neuroblastoma.^38^ 4‐chlorophenylacetic is correlated with intestinal flora.^39^ Recent studies have shown that calcium intake and malnutrition can lead to outbreaks of SMA and changes in the gut microbiota are associated with its severity and progression.^40^ Our study showed that 4‐chlorophenylacetic acid was significantly and negatively associated with HFMSE and could be used as a marker for SMA classification. One possible explanation is that patients with SMA type II generally have a poorer nutritional status than patients with SMA type III, which leads to changes in the gut flora that ultimately manifest as differences in the levels of 4‐chlorophenylacetic acid.

Sodium dodecyl benzenesulfonate is a ligand for peroxisome proliferator‐activated receptor γ.^41^ Dodecyl benzenesulfonic acid can be used as a serum metabolite biomarker for colorectal cancer^42^ and nonproliferative diabetic retinopathy.^43^ Conductive hydrogel composites containing dodecyl benzenesulfonic acid promote neurite growth and are, therefore, promising candidates for nerve regeneration.^44^ Our results suggest significantly higher levels of dodecyl benzenesulfonic acid in the CSF of patients with SMA type II than in that of those with type III, further supporting the hypothesis that dodecyl benzenesulfonic acid promotes nerve growth. Therefore, dodecyl benzenesulfonic acid may be an objective predictor of SMA efficacy.

Cinobufagin is a well‐known Chinese medicine obtained from the dried skin of toads. It is widely used to treat various cancers.^45^ Cinobufagin induces cell‐cycle arrest at the S phase^46^ and promotes apoptosis in human glioblastoma cells.^47^ Our results showed decreased levels of cinobufagin in the CSF of patients with SMA type III, consistent with lower motor neuron apoptosis in patients with SMA type III than in those with SMA type II. However, the results of the correlation analysis showed that cinobufagin was positively correlated with HFMSE, which seemed to be contradictory. One possible explanation is that patients with SMA exhibit inflammatory features^48^ and cinobufagin can modulate the innate immune response in humans and trigger antimicrobial activity.^49^

The proportion of compound heterozygous mutations was about 17.5%, which may be due to selection bias because of the small number of cases (40) included in this study. Studies of patients with SMA having compound heterozygous mutations have shown that the severity of their clinical phenotype is primarily related to the type and location of the mutations.^50^, ^51^, ^52^, ^53^ However, to date, a few studies have investigated whether genetic phenotype has an effect on CSF metabolic parameters. Here, we grouped patients with homozygous deletion and compound heterozygous mutations in terms of clinical phenotypes and analyzed their respective differentially expressed metabolites, which revealed four metabolites that were unaffected by gene phenotype and appeared to be more suitable candidates for use as molecular markers for predicting the clinical prognosis of SMA. Among them, N‐myristoyl arginine is a member of the class of compounds called N‐acyl amides. These molecules have a fatty acyl group attached to arginine, and they have been shown to play a role in cell migration, inflammation, and pathologies, including diabetes, cancer, neurodegenerative disease, and obesity.^54^, ^55^, ^56^

This study identified metabolite markers in the CSF of children with SMA types II and III. However, this study has some limitations that warrant consideration. Although we identified promising metabolic typing markers, further external validation is required to assess the sensitivity and specificity of these biomarkers. More specifically, we plan to analyze CSF metabolites using external data in the future to better characterize the metabolic profile and predict SMA classification. Although we have a reasonable explanation for the utility of some of the differential metabolites, some metabolic markers lack reasonable explanations owing to limited research, thereby presenting directions for future research. Finally, changes in metabolite levels may be influenced by diet and nutritional status; these confounding factors should be excluded in future studies.

## 5 CONCLUSIONS

To summarize, we have demonstrated metabolic markers in CSF associated with the classification of SMA in children, including 4‐chlorophenylacetic acid, sodium dodecyl benzenesulfonate, cinobufagin, and N‐myristoyl arginine. These metabolic markers in the CSF are promising candidate prognostic factors for SMA.

We also identified the metabolic pathways associated with the severity of SMA, including lysine degradation, arginine metabolism, and tyrosine metabolism. The results of the present study enhance our understanding of SMA.

## AUTHOR CONTRIBUTIONS

All authors contributed to the manuscript. Mengnan Lu contributed to manuscript writing, data collection, and analysis. Dan Li designed the study. Xueying Wang collected cerebrospinal fluid samples. Na Sun recorded the clinical data. Lin Yang and Shaoping Huang supervised the revision of the manuscript.

## FUNDING INFORMATION

This study was funded by the Key Research and Development Program of Shaanxi [No. 2021SF‐087].

## CONFLICT OF INTEREST STATEMENT

The authors declare no conflict of interest.

## ETHICS STATEMENT

This study was approved by the Ethics Committee of the Xi'an Jiaotong University Second Affiliated Hospital (2022 Ethics Approval—Research No. 038).

## PATIENT CONSENT

Families of all participants included in this study provided informed consent, as did children older than 8 years of age, all of whom were given a comprehensive explanation about the study.

## Supporting information


Supplementary Information S1.

Supplementary Information S2.

Supplementary Information S3.

Supplementary Information S4.

## Data Availability

The data that support the findings of this study are available from the corresponding author upon reasonable request.
